# Efficacy and Safety of Luseogliflozin (TS-071) in Japanese Children and Adolescents with Type 2 Diabetes Mellitus: A Multicenter, Randomized, Double-Masked, Placebo-Controlled Phase 3 Study

**DOI:** 10.1159/hrp/adaag009

**Published:** 2026-09-05

**Authors:** Toru Kikuchi, Hidefumi Nakamura, Hideo Umeuchi, Iwao Kitajima, Hironori Yamasaki, Tatsuhiko Urakami

**Affiliations:** a Department of Pediatrics, Saitama Medical University Hospital, Saitama, Japan; b Department of Research and Development Supervision, National Center for Child Health and Development, Tokyo, Japan; c Development Headquarters, Taisho Pharmaceutical Co. Ltd, Tokyo, Japan; d Department of Pediatrics, Nihon University Hospital, Tokyo, Japan

**Keywords:** Pediatric patients, Luseogliflozin, Efficacy, Safety, Type 2 diabetes

## Abstract

**Introduction:**

This study evaluated the efficacy and safety of luseogliflozin (TS-071), a sodium-glucose cotransporter 2 inhibitor, in Japanese children and adolescents with type 2 diabetes (T2D).

**Methods:**

In this multicenter, randomized, double-masked, placebo-controlled phase 3 trial, patients aged 10–17 years with hemoglobin A1c (HbA1c) levels of 7.0%–12.0%, with or without prior metformin use, were enrolled. Participants were randomized (2:1) to receive luseogliflozin 2.5 mg or placebo for 12 weeks (period I), followed by a 40-week open-label extension (period II), allowing dose escalation to 5 mg.

**Results:**

Forty-eight participants were randomized and received luseogliflozin (*n* = 31) or placebo (*n* = 17). The primary endpoint – change in HbA1c from baseline at the end of period I – was −1.45% (95% CI, −1.78% to −1.11%) in the luseogliflozin group. The between-group difference in HbA1c change was −1.76% (95% CI, −2.49% to −1.02%). Luseogliflozin maintained efficacy through 52 weeks, with a mean HbA1c change of −0.92% ± 1.74% at the end of period II. Adverse events (AEs) and adverse drug reactions (ADRs) occurred in 85.4% and 29.2% of patients, respectively. No deaths occurred; three serious AEs and one mild hypoglycemic event were reported. Urinary and genital infections were the most frequent ADRs.

**Conclusion:**

Luseogliflozin significantly improved glycemic control and was well tolerated, supporting its potential as an effective and safe treatment option for children and adolescents with T2D.

## Introduction

Type 2 diabetes (T2D) arises from inadequate insulin action due to pancreatic β-cell dysfunction along with insulin resistance caused by excess visceral adiposity and obesity [1, 2]. The global rise in childhood obesity parallels the increasing incidence of T2D among children and adolescents [3]. In the USA, the incidence of pediatric T2D increased by an average of 4.8% annually between 2002 and 2015 [4], affecting approximately 28,000 children by 2017 [5]. If this upward trend continues, the number of children and adolescents with T2D may reach 220,000 by 2060, highlighting a serious public health concern. A global prevalence study in 2021 also identified China and India, along with the USA, as countries with the highest estimated rates of pediatric T2D, indicating an expanding disease burden in Asia [3]. In Japan, however, recent data on temporal trends in pediatric T2D incidence remain unclear.

Japan has implemented nationwide urine screening programs for diabetes since 1992, encompassing schoolchildren from elementary through high school. The annual detection rate of T2D is 0.80 per 100,000 elementary school children and 6.41 per 100,000 junior high school students [6]. Because childhood-onset type 1 diabetes (T1D) occurs in 2.25 per 100,000 Japanese junior high school students, T2D is more prevalent in this age group than T1D [7]. Approximately 80% of Japanese children and adolescents with T2D are obese, and over 50% have a family history of disease in T2D.

The Japan Diabetes Society (JDS) and the Japanese Society for Pediatric Endocrinology, consistent with recommendations from the International Society for Pediatric and Adolescent Diabetes (ISPAD) [8] and the American Diabetes Association (ADA) [9], advocate for initial management of pediatric T2D through diet, exercise, and behavioral modification. However, achieving adequate glycemic control – defined as hemoglobin A1c (HbA1c) <7.0% – through lifestyle intervention alone is often insufficient, necessitating pharmacologic treatment. In Japan, only metformin [10] and glimepiride are currently approved oral antidiabetic agents for pediatric use. Due to the high prevalence of obesity among Japanese children and adolescents with T2D, sulfonylureas such as glimepiride are used infrequently, with metformin monotherapy being the predominant approach. Nevertheless, studies such as the TODAY trial have shown that metformin alone does not always achieve satisfactory glycemic control [11]. Recently, the US Food and Drug Administration and the European Medicines Agency have approved sodium-glucose cotransporter 2 (SGLT2) inhibitors and glucagon-like peptide-1 receptor agonists for pediatric T2D following positive outcomes from phase 3 clinical trials [12–14]. In Japan, expanding the availability of pediatric antidiabetic agents remains a key unmet need.

Luseogliflozin (TS-071), a selective SGLT2 inhibitor, is approved for adult T2D treatment in Japan. Considering that the pathophysiology of T2D in children and adolescents closely parallels that in adults, luseogliflozin is expected to provide similar glycemic benefits in younger populations. A pharmacokinetic study in pediatric patients with T2D demonstrated that the pharmacokinetic parameters of luseogliflozin – including C_max_, t_max_, AUC_0–∞_, t_1/2_, and urinary glucose excretion – were comparable to those observed in adults [15]. Unlike current ISPAD and ADA guidelines, which generally position SGLT2 inhibitors as add-on therapies to insulin or metformin when glycemic targets are not achieved, this study did not require background insulin or metformin therapy but rather was intended for evaluating luseogliflozin as an early pharmacological treatment option, including its potential for first-line use. In this setting, this study aimed to evaluate the efficacy and safety of luseogliflozin in children and adolescents with T2D using the same dosage and administration regimen approved for adults in Japan.

## Methods

### Study Design

This multicenter, randomized, double-masked, placebo-controlled, phase 3 trial was conducted at 58 institutions across Japan between March 2021 and March 2025. The study was registered on December 15, 2020, with the Japan Registry of Clinical Trials (jRCT2031200244; https://jrct.mhlw.go.jp/latest-detail/jRCT2031200244). It adhered to the principles of the Declaration of Helsinki, Japanese Pharmaceutical Affairs Law, and Good Clinical Practice guidelines. The protocol was approved by the Institutional Review Board of each participating institution (online suppl. Table 1; for all online suppl. material, see https://doi.org/10.1159/hrp/adaag009). The CONSORT 2025 checklist is provided as online supplementary material. Written informed consent was obtained from the parents or legal guardians of all participants, and assent was obtained from all pediatric participants before enrollment.

### Eligibility Criteria

Online supplementary information summarizes the study inclusion and exclusion criteria. Eligible participants were Japanese outpatients aged 10–17 years diagnosed with T2D according to the JDS guidelines [16]. Inclusion criteria required an HbA1c level between 7.0% and 12.0% at week −4, with all HbA1c values from week −12 to week −4 remaining within ±1.0% of the week −4 value. A standardized uniform diet or exercise therapy across all participants was not specified; individualized pre-study regimens were continued to facilitate appropriate luseogliflozin efficacy evaluation. Investigators confirmed and recorded diet and exercise therapy adherence through patient interviews at each study visit using the electronic data capture system. Participants were required to maintain consistent dietary therapy for at least 8 weeks before week −4. Although exercise and metformin therapy were not mandatory, participants engaging in these treatments were required to maintain stable regimens during the study. For participants receiving metformin, the dose was required to be within the approved dosage range in Japan (500–2,000 mg/day or up to 2,250 mg/day for ≥15-year-old participants), administered in two or three divided doses. The following were the exclusion criteria: patients who had diabetes types other than T2D (including TID, monogenic, or secondary diabetes); positivity for pancreatic islet-related autoantibodies; endocrine or genetic disorders affecting glycemic control; prior use of antidiabetic medications other than metformin; significant renal, hepatic, cardiovascular, or other systemic diseases; unstable psychiatric disorders; or other conditions associated with increased safety risk. Recurrent diabetic ketoacidosis, advanced diabetic complications, active or prior malignancy, and pregnancy encompassed additional exclusions. The study protocol did not mandate autoimmune antibody testing and other definitive diagnostic evaluations, which were performed at the investigator’s discretion based on routine clinical practice.

### Interventions and Procedures

Participants received oral, once-daily luseogliflozin before breakfast, in addition to standard care (diet, exercise, or metformin therapy). Following a 4-week lead-in period, participants were randomized (2:1) using an Interactive Web Response System to receive luseogliflozin 2.5 mg or placebo for 12 weeks (period I). Randomization was stratified by HbA1c at week −4 (≥7.0% to <7.5%, ≥7.5% to <8.0%, and ≥8.0% to ≤12.0%) and age at consent (≥10 to <15 years and ≥15 to <18 years). Treatment allocation was kept strictly confidential in this clinical study, accessible only to the allocation staff and the emergency key code managers in this clinical study until database lock. All investigational drugs and their packaging were manufactured by Taisho Pharmaceutical to be identical in appearance. After period I, all participants entered a 40-week open-label extension (period II) with luseogliflozin 2.5 mg for long-term safety follow-up. Dose escalation to 5 mg was permitted at weeks 24, 28, or 36 if HbA1c levels at the two most recent visits were ≥7.5% and the investigator determined no safety concerns (Fig. 1). Assessments occurred at week −4 (lead-in), day 1 (baseline), weeks 4, 8, and 12 (period I), and weeks 16, 20, 24, 28, 36, 44, and 52 (period II).

**Fig. 1.**
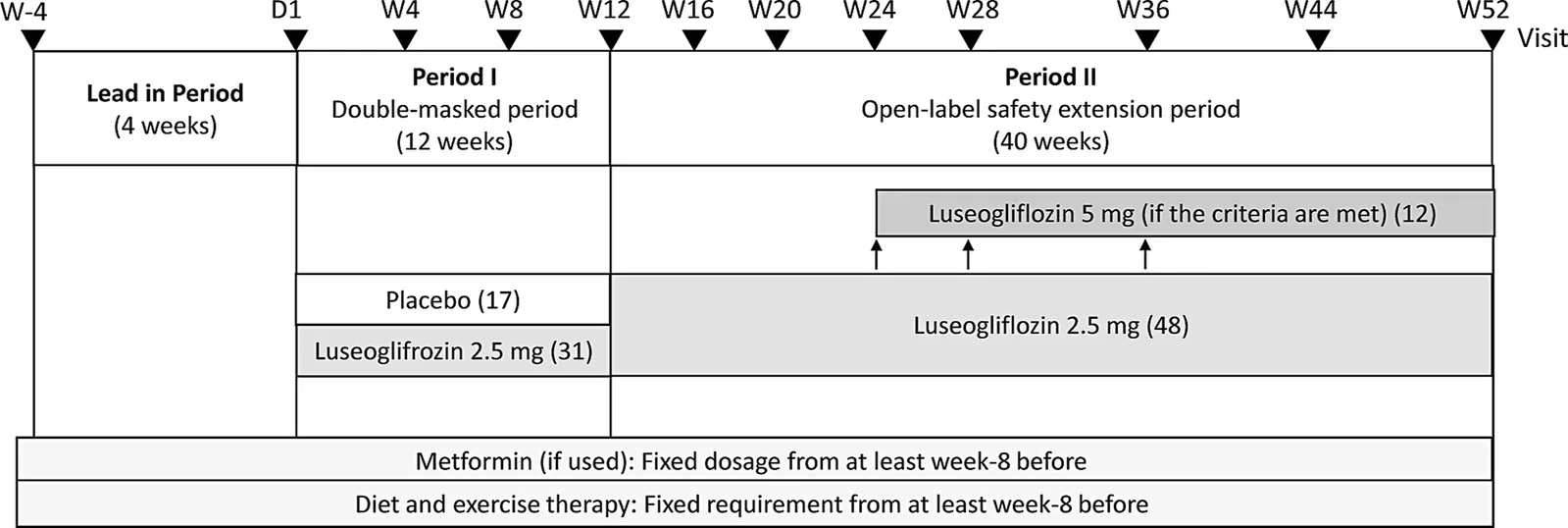
Study design. The schematic representation of the study design indicates the visit schedule and dosing regimen at each phase. The study comprised a 4-week lead-in period, 12-week double-masked treatment period (period I), and 40-week open-label safety extension period (period II). During period I, participants received placebo or luseogliflozin 2.5 mg. During period II, all participants received luseogliflozin 2.5 mg, with dose escalation to 5 mg based on predefined criteria at either week 24, 28, or 36. Black arrows denote the time points at which dose escalation may be implemented.

### Outcome Measures

The primary efficacy endpoint was the mean change in HbA1c from baseline to week 12 at the end of period I with luseogliflozin 2.5 mg. The key secondary endpoint compared the mean change in HbA1c from baseline to week 12 between the luseogliflozin 2.5 mg and placebo groups. The secondary endpoint was the change in HbA1c at week 52 following long-term treatment. Other secondary efficacy endpoints included fasting plasma glucose (FPG) and body weight at week 12. Exploratory endpoints included glycated albumin (GA), fasting plasma insulin, fasting plasma C-peptide, fasting serum lipids, homeostatic model assessment for insulin resistance (HOMA-R), homeostatic model assessment of β-cell function, and percent overweight (POW [%] = 100 × [measured body weight − standard body weight]/standard body weight) at week 12.

Safety was assessed using pooled data from luseogliflozin 2.5 mg and 5 mg across both periods, based on the incidence of adverse events (AEs), adverse drug reactions (ADRs), serious AEs (SAEs). Moreover, AEs and ADRs of special interest were evaluated; these categories are predefined in online supplementary Table 2 and encompass known or potential risks related to SGLT2 inhibition, including hypoglycemia, pollakiuria, volume depletion, ketoacidosis, urinary and genital infections, decreased blood pressure, tumors, cardiovascular events, bone metabolism effects, renal impairment, hepatic dysfunction, and gastrointestinal disorders. During period I, safety assessment involved a comparison of AEs between luseogliflozin 2.5 mg and placebo.

All AEs were coded according to the Japanese version of the Medical Dictionary for Regulatory Activities (MedDRA/J ver. 27.0) and classified by organ system and preferred term. Investigators evaluated each AE for causality (drug-related or not) and severity (mild, moderate, or severe), recording details in patient case report forms.

### Sample Size

Sample size estimation was based on expected changes in HbA1c from prior adult studies (TS071-03-2, TS071-03-3, and TS071-03-5). To achieve 80% power with a two-sided 95% confidence interval (CI) upper limit <0.00%, 27 participants were required in the luseogliflozin 2.5 mg group. Assuming a 2:1 randomization ratio and 10% dropout rate, the total target enrollment was 45 participants (30 in the luseogliflozin 2.5 mg group and 15 in the placebo group).

### Statistical Analysis

Efficacy was evaluated using the full analysis set, which included all participants who received at least one dose of the study drug and had at least one post-baseline efficacy evaluation. Safety analyses used the safety analysis set, comprising all participants who received at least one dose and had safety data posttreatment.

For the primary endpoint, unadjusted CIs were computed using the t-distribution. For secondary endpoints, within-group changes and between-group differences were estimated using a model adjusting for baseline values and age. No multiplicity adjustments were made. If week 12 or week 52 data were missing or invalid, the last observation carried forward method was applied. Statistical analyses were conducted using SAS version 9.4 (SAS Institute Japan).

## Results

### Study Sample

Of the 76 participants screened, 49 were randomized to either the luseogliflozin 2.5 mg group (*n* = 32) or the placebo group (*n* = 17) (Fig. 2). Twenty-seven participants were not randomized because of failure to meet the inclusion or exclusion criteria. One participant in the luseogliflozin 2.5 mg group was withdrawn after randomization because of a protocol violation before dosing.

**Fig. 2.**
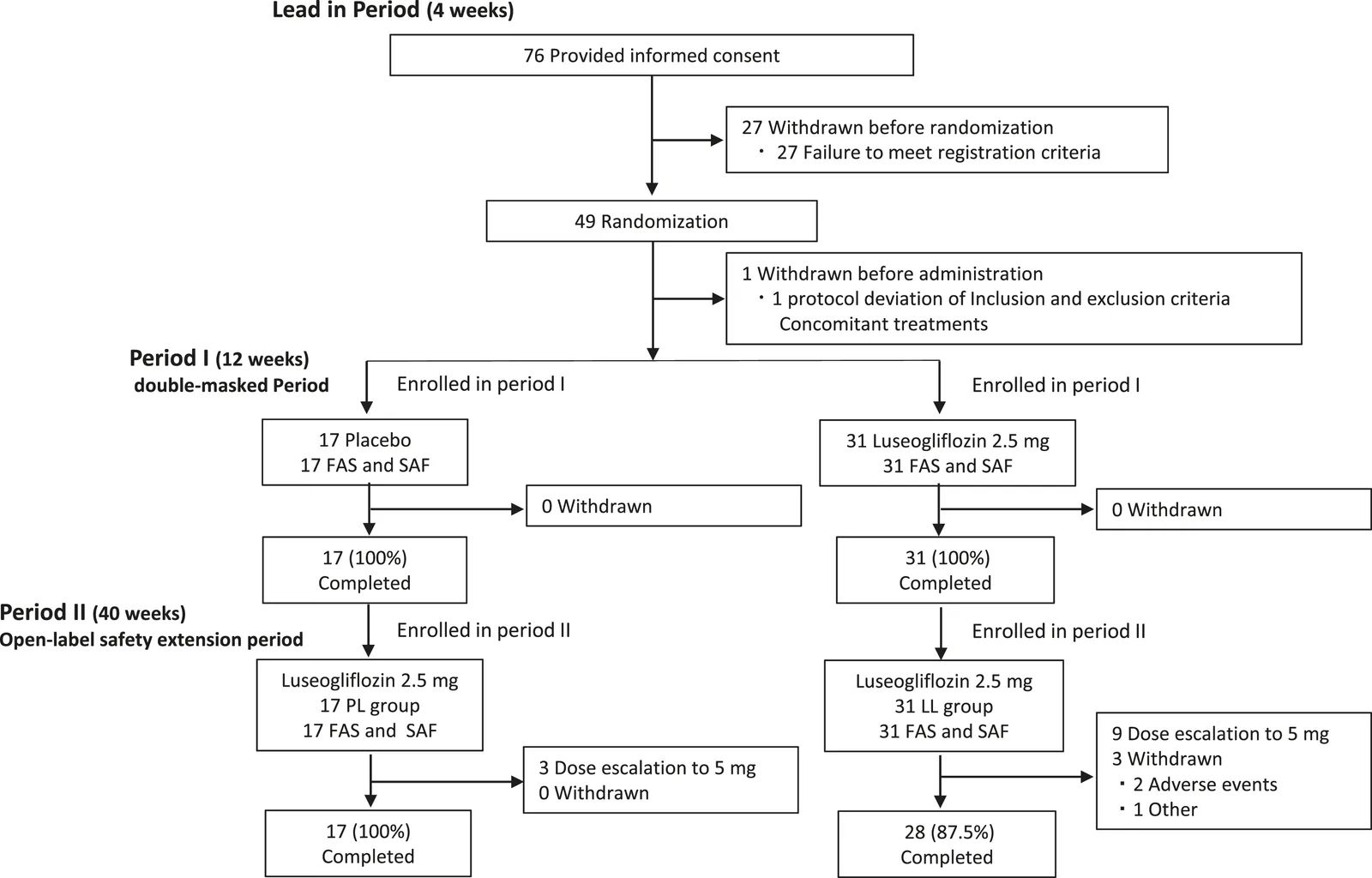
Patient distribution. The diagram shows the flow of patient enrollment, randomization, treatment allocation, and analysis sets. Of the 76 patients who provided informed consent, 49 were randomized to the luseogliflozin 2.5 mg group (*n* = 31) or the placebo group (*n* = 17). One patient was withdrawn before administration because of protocol deviation. The full analysis set and safety analysis set are shown for each group. Completion rates and dose escalation during period II are also indicated. LL, luseogliflozin-to-luseogliflozin; PL, placebo-to-luseogliflozin.

All 48 treated participants completed period I and entered period II. During period II, three participants in the luseogliflozin-to-luseogliflozin (LL) group discontinued – two because of AEs and one because of insufficient therapeutic effect. No discontinuations occurred in the placebo-to-luseogliflozin (PL) group. Dose escalation to luseogliflozin 5 mg was implemented in 12 participants (9 in the LL group and 3 in the PL group). All 48 participants were included in the full analysis set and safety analysis set for both period I and the overall analysis (periods I and II).

### Demographics and Baseline Characteristics

Table 1 shows the demographic and baseline characteristics of all 48 participants. All enrolled participants were Japanese and classified as Asian. No clinically meaningful differences in demographic or other baseline characteristics were observed between the groups. Of the 48 participants, 15 (31.3%) were male and 33 (68.8%) were female, showing female predominance. The mean age, body weight, BMI POW, and duration of disease were 14.3 years, 74.61 kg, 28.74 kg/m^2^, 46.04%, and 2.13 years, respectively. At baseline, mean HbA1c, FPG, and HOMA-R were 8.87%, 187.4 mg/dL, and 8.98, respectively. Thirty-three participants (68.8%) were receiving concomitant metformin therapy. Throughout this study, diet and exercise therapy adherence was generally maintained, and no protocol deviations associated with concomitant metformin therapy nonadherence were reported.

**Table 1.** Demographic and other baseline characteristics (full analysis set)

Dose (*n*)	Placebo (17)	Luseogliflozin 2.5 mg (31)	Total (48)
Sex (male:female), *n* (%)	8 (47.1):9 (52.9)	7 (22.6):24 (77.4)	15 (31.3):33 (68.8)
Age (min, max), years	14.3±1.4 (12, 17)	14.4±1.8 (11, 17)	14.3±1.7 (11, 17)
Diagnosis age (min, max), years	12.6±1.2 (10, 15)	12.1±1.7 (9, 16)	12.3±1.6 (9, 16)
Disease duration, years	1.71±1.25	2.36±1.81	2.13±1.65
Body weight, kg	76.59±20.69	73.53±17.29	74.61±18.41
BMI, kg/m^2^	29.61±5.19	28.26±5.49	28.74±5.37
BMI-SDS	1.943±0.707	1.732±1.156	1.805±1.019
POW, %	52.76±22.58	42.35±26.19	46.04±25.24
eGFR, mL/min/1.73 m^2^	142.59±34.07	142.22±30.46	142.35±31.42
Diabetes complication, *n* (%)	1 (5.9)	0	1 (2.1)
HbA1c, %	9.01±1.35	8.79±1.04	8.87±1.15
FPG, mg/dL	190.9±56.2	185.5±42.6	187.4±47.4
HOMA-R	9.12±3.80	8.89±6.08	8.98±5.34
Metformin (nonuse:use), *n*	6:11	9:22	15:33
Metformin dose (*n*), mg/day	1,227.3±480.3 (11)	1,147.7±605.8 (22)	1,174.2±560.7 (33)

Values are presented as means ± SDs.

BMI, body mass index; BMI-SDS, BMI standard deviation score; POW, percent overweight; eGFR, estimated glomerular filtration rate; HbA1c, hemoglobin A1c; FPG, fasting plasma glucose; HOMA-R, homeostatic model assessment for insulin resistance; SD, standard deviation.

### Efficacy

#### Primary and Key Secondary Endpoints

For the primary endpoint, the mean change in HbA1c from baseline to week 12 at the end of period I in the luseogliflozin group was −1.45% (95% CI, −1.78% to −1.11%), demonstrating a significant reduction (Table 2, Fig. 3). The between-group difference in HbA1c change (key secondary endpoint) at week 12 was −1.76% (95% CI, −2.49% to −1.02%) in favor of luseogliflozin. HbA1c levels decreased by week 4, continued to decline through week 16, and remained stable up to week 52 in the LL group (including participants escalated to 5 mg). At week 52 (end of study), the mean (±standard deviation) change from baseline was −0.92% ± 1.74%, confirming sustained glycemic improvement (Fig. 4, online suppl. Table 3).

**Table 2.** Primary and key secondary outcomes for HbA1c (full analysis set)

Luseogliflozin dose (*n*)	HbA1c (%)				
	baseline mean±SD	end of period I			
		mean±SD	change from baseline mean±SD	primary outcome	key secondary outcome
				change from baseline mean [95% CI]	difference from placebo point estimate [95% CI]
Placebo (17)	9.01±1.35	9.22±1.82	0.21±1.81	​	−1.76 [−2.49, −1.02]
2.5 mg (31)	8.79±1.04	7.35±0.98	−1.45±0.92	−1.45 [−1.78, −1.11]	

HbA1c, hemoglobin A1c; SD, standard deviation; CI, confidence interval.

**Fig. 3.**
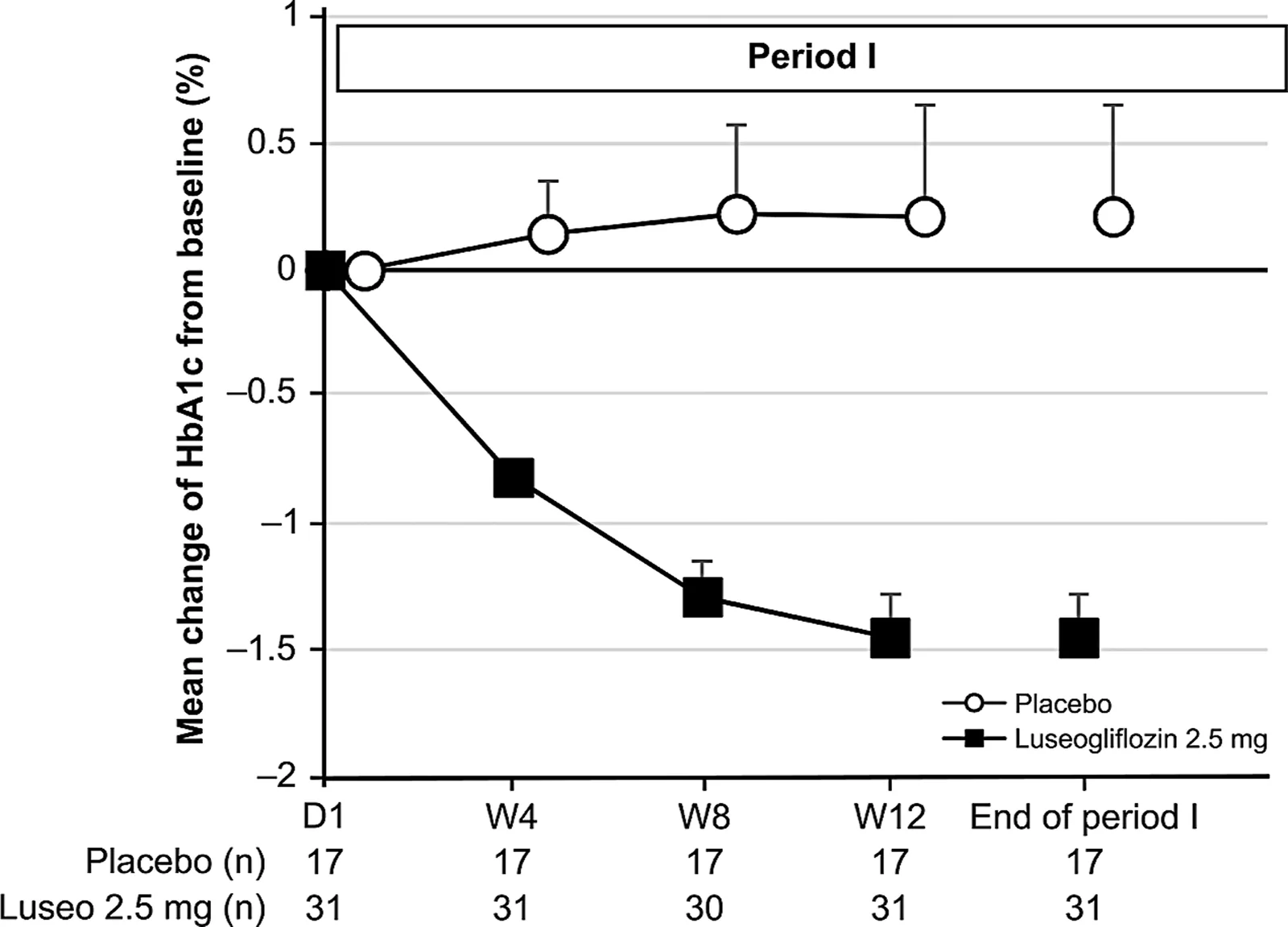
Change in HbA1c from baseline to week 12 (period I) (full analysis set). Mean change in HbA1c (%) from baseline to week 12 in the placebo and luseogliflozin 2.5 mg groups in the full analysis set population. Data are presented as means ± SE. Values at the end of period I were imputed using the last observation carried forward method.

**Fig. 4.**
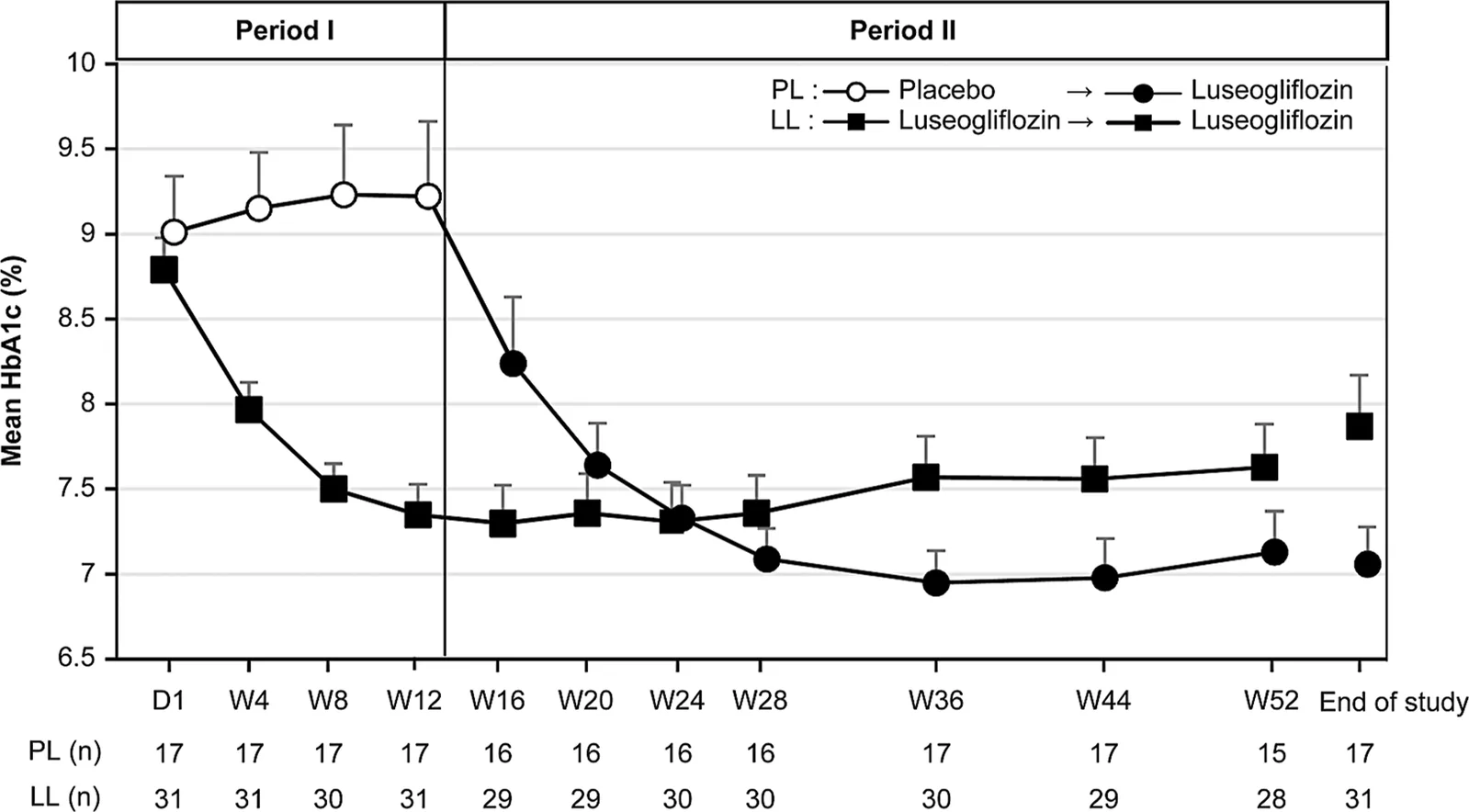
Mean HbA1c from baseline to week 52 (periods I and II) (full analysis set). Mean HbA1c from baseline to week 52 in the PL and LL groups in the full analysis set population. Data are presented as means ± SE. Values at the end of study were imputed using the last observation carried forward method.

#### Secondary and Exploratory Endpoints

Between-group comparisons of secondary and exploratory outcomes at week 12 are presented in Table 3. Among secondary endpoints, FPG reduction was significantly greater in the luseogliflozin group (−54.3 mg/dL) than in the placebo group (−9.9 mg/dL), yielding a between-group difference of −44.3 mg/dL (95% CI, −67.3 mg/dL to −21.4 mg/dL). Among exploratory endpoints, GA and HOMA-R showed significant improvements in the luseogliflozin group relative to baseline and to the placebo group. Changes in other exploratory parameters were minimal and not statistically significant.

**Table 3.** Secondary and exploratory endpoints (full analysis set)

Luseogliflozin dose	*n*	Baseline mean±SD	End of period I mean±SD	Change from baseline point estimate [95% CI]	Difference from placebo point estimate [95% CI]
*Secondary endpoints*					
FPG (mg/dL)					
Placebo	17	190.9±56.2	178.5±52.8	−9.9 [−28.4, 8.5]	−44.3 [−67.3, −21.4]
2.5 mg	31	185.5±42.6	132.6±30.9	−54.3 [−67.9, −40.6]	
Body weight (kg)					
Placebo	17	76.59±20.69	76.05±21.01	−0.51 [−1.55, 0.52]	−0.44 [−1.73, 0.84]
2.5 mg	31	73.53±17.29	72.58±16.95	−0.96 [−1.72, −0.19]	
*Exploratory endpoints*					
GA (%)					
Placebo	17	20.82±4.23	20.69±5.60	0.11 [−1.69, 1.91]	−4.74 [−6.99, −2.49]
2.5 mg	31	19.87±3.48	15.36±3.36	−4.64 [−5.97, −3.30]	
Fasting insulin (μU/mL)					
Placebo	17	20.35±9.47	17.86±11.70	−2.31 [−5.27, 0.65]	−0.29 [−3.98, 3.39]
2.5 mg	31	19.03±12.74	16.52±10.24	−2.60 [−4.79, −0.41]	
Fasting C-peptide (ng/mL)					
Placebo	17	3.39±0.85	3.07±1.08	−0.34 [−0.66, −0.02]	0.07 [−0.33, 0.47]
2.5 mg	31	3.59±1.51	3.31±1.46	−0.27 [−0.51, −0.03]	
HOMA-R					
Placebo	17	9.12±3.80	7.85±5.26	−1.20 [−2.64, 0.23]	−2.32 [−4.10, −0.53]
2.5 mg	31	8.89±6.08	5.41±3.33	−3.52 [−4.58, −2.46]	
HOMA-β (%)					
Placebo	17	70.16±45.65	90.08±122.92	18.89 [−19.89, 57.67]	21.38 [−26.96, 69.72]
2.5 mg	31	61.03±45.80	100.75±72.74	40.27 [11.60, 68.95]	
Total cholesterol (mg/dL)					
Placebo	17	200.4±40.1	192.5±36.2	−6.9 [−17.1, 3.3]	12.8 [0.1, 25.5]
2.5 mg	31	188.5±38.8	194.9±41.7	5.9 [−1.6, 13.4]	
LDL cholesterol (mg/dL)					
Placebo	17	129.2±37.3	126.2±35.0	−2.7 [−11.0, 5.6]	7.4 [−2.9, 17.7]
2.5 mg	31	124.2±32.4	129.0±35.6	4.7 [−1.5, 10.8]	
HDL cholesterol (mg/dL)					
Placebo	17	51.2±10.5	48.4±8.2	−2.7 [−5.6, 0.2]	4.9 [1.3, 8.6]
2.5 mg	31	48.8±14.8	51.1±15.3	2.2 [0.1, 4.4]	
Triglycerides (mg/dL)					
Placebo	17	142.6±82.2	143.8±67.1	2.6 [−30.3, 35.5]	−12.2 [−53.1, 28.8]
2.5 mg	31	134.4±67.1	125.6±93.8	−9.6 [−33.9, 14.8]	
BMI					
Placebo	17	29.61±5.19	29.41±5.47	−0.20 [−0.59, 0.19]	−0.22 [−0.70, 0.26]
2.5 mg	31	28.26±5.49	27.84±5.44	−0.42 [−0.70, −0.13]	
BMI-SDS^a^					
Placebo	16	1.943±0.707	1.888±0.792	−0.059 [−0.147, 0.028]	−0.035 [−0.146, 0.075]
2.5 mg	28	1.732±1.156	1.635±1.235	−0.095 [−0.161, −0.029]	
POW (%)					
Placebo	17	52.76±22.58	51.81±24.43	−1.00 [−3.03, 1.03]	−1.23 [−3.78, 1.31]
2.5 mg	31	42.35±26.19	40.09±26.13	−2.24 [−3.73, −0.74]	

FPG, fasting plasma glucose; LDL, low-density lipoprotein; HDL, high-density lipoprotein; HOMA-R, homeostatic model assessment for insulin resistance; HOMA-β, homeostatic model assessment of β-cell function; BMI, body mass index; BMI-SDS, BMI standard deviation score; POW, percent overweight; SD, standard deviation; CI, confidence interval.

^a^ The calculation excluded participants aged ≥17 years and 7 months.

#### Subgroup Analysis

Subgroup analyses by sex, age, BMI, metformin use, and baseline HbA1c demonstrated that HbA1c reduction was greater in male participants and those with higher baseline HbA1c levels (Table 4). These differences likely reflected higher initial HbA1c values rather than intrinsic variation in drug response, suggesting consistent efficacy across subgroups.

**Table 4.** Subgroup-specific changes in HbA1c at the end of treatment period I in the luseogliflozin 2.5 mg group (full analysis set)

Subgroup	Category	*n*	HbA1c (%) (mean±SD)		
			baseline	end of period I	change from baseline
Age, years	<15	17	8.94±1.13	7.27±1.11	−1.66±0.93
	≥15	14	8.62±0.93	7.44±0.83	−1.18±0.87
Sex	Male	7	9.40±1.06	7.54±0.93	−1.86±0.75
	Female	24	8.62±0.98	7.29±1.01	−1.33±0.95
HbA1c (%)	<7.5	2	NC	NC	NC
	7.5 to <8.5	11	7.98±0.31	6.81±0.53	−1.17±0.42
	≥8.5	18	9.48±0.73	7.77±1.04	−1.72±1.07
Metformin use	No	9	8.72±0.96	7.27±0.71	−1.46±0.57
	Yes	22	8.82±1.09	7.38±1.09	−1.44±1.05
BMI	<25.0	7	8.30±0.79	6.91±1.02	−1.39±0.52
	≥25.0	24	8.94±1.07	7.48±0.96	−1.46±1.02

NC, not calculated due to small sample size (*n* ≤2); HbA1c, hemoglobin A1c; BMI, body mass index; SD, standard deviation.

### Safety

During period I, AEs occurred in 71.0% (22 of 31) in the luseogliflozin 2.5 mg group and 52.9% (9 of 17) in the placebo group (Table 5). ADRs were reported only in the luseogliflozin 2.5 mg group (29.0%). Across periods I and II (excluding the placebo phase in the PL group), AEs were reported in 85.4% (41 of 48) of the participants, and drug-related AEs in 29.2% (14 of 48). The most common AEs (≥5% of participants) were nasopharyngitis, immune reactions (postvaccination reactions to COVID-19 vaccines), influenza, COVID-19, allergic rhinitis, diarrhea, cystitis, and headache. No deaths occurred during the study.

**Table 5.** Summary of AEs and drug-related AEs during the study period (safety analysis set)

Period	Entire period^a^	Period I		After dose escalation in period II
Dose (*n*)	luseogliflozin (48)	placebo (17)	luseogliflozin 2.5 mg (31)	luseogliflozin 5 mg (12)
	% (*n*)	% (*n*)	% (*n*)	% (*n*)
AEs (%)	85.4 (41)	52.9 (9)	71.0 (22)	50.0 (6)
ADRs (%)	29.2 (14)	0	29.0 (9)	16.7 (2)
Fatal	0	0	0	0
SAE (%)	6.3 (3)	0	0	0
AEs leading to withdrawal	4.2 (2)	0	0	0
Common AEs (%)^b^	​	​	​	​
Diarrhea	8.3 (4)	0	9.7 (3)	0
Cystitis	6.3 (3)	0	3.2 (1)	0
Influenza	10.4 (5)	5.9 (1)	6.5 (2)	0
Nasopharyngitis	22.9 (11)	11.8 (2)	12.9 (4)	0
COVID-19	10.4 (5)	0	0	8.3 (1)
Immunization reaction	14.6 (7)	5.9 (1)	3.2 (1)	8.3 (1)
Headache	6.3 (3)	0	0	0
Rhinitis allergic	10.4 (5)	0	3.2 (1)	8.3 (1)

AEs, adverse events; ADRs, adverse drug reactions; SAE, severe adverse event.

^a^ AEs occurring after dose escalation to luseogliflozin 5 mg were assessed across the entire study period (periods I and II), excluding those that occurred during placebo administration in the PL group (period I).

^b^ AEs reported in ≥5% of patients across the entire period were classified as common AEs.

Three SAEs were reported – constipation, diabetes mellitus (worsening of glycemic control), and Fournier’s gangrene, each in separate participants. Constipation and Fournier’s gangrene were drug-related. Diabetes mellitus and Fournier’s gangrene resulted in treatment discontinuation for two participants. One participant required temporary dose interruption due to COVID-19 infection.

The participant who developed Fournier’s gangrene was a 13-year-old Japanese female with a baseline HbA1c of 8.3%, not receiving metformin. BMI was within the normal range at 23.9 kg/m^2^, and abdominal pain was the only reported comorbidity. Baseline laboratory findings of inflammatory-related parameters showed abnormalities, including fungal elements in the urinary sediment, which were mild. Therefore, no definitive contributing factors for Fournier’s gangrene were identified based on the patient background.

After dose escalation to 5 mg, AEs occurred in 50.0% (6 of 12) of participants, and ADRs in 16.7% (2 of 12). The incidence and nature of AEs and ADRs after dose escalation were comparable to those observed overall, with no new or unexpected safety signals (Table 5).

AEs and ADRs of special interest are summarized in Table 6. One mild case of hypoglycemia occurred and did not require treatment discontinuation. Urinary tract and genital infections occurred relatively frequently but were consistent with known class effects of SGLT2 inhibitors. No cases of ketoacidosis were observed, although some participants exhibited transient elevations in ketone body levels (acetoacetic acid and β-hydroxybutyric acid). Blood and urinary ketone measurements were performed during study visits, and additional ketone or blood gas assessments were conducted when clinically indicated. No clinically significant changes were noted in other laboratory parameters (online suppl. Table 4).

**Table 6.** AEs and ADRs of special interest (safety analysis set)

Categories	Entire period^a^ (48)	
	AEs of special interest, % (*n*)	ADRs of special interest, % (*n*)
Related to hypoglycemia	2.1 (1)	2.1 (1)
Hypoglycemia	2.1 (1)	2.1 (1)
Related to pollakiuria	8.3 (4)	6.3 (3)
Enuresis	2.1 (1)	0
Pollakiuria	2.1 (1)	2.1 (1)
Polyuria	4.2 (2)	4.2 (2)
Related to ketoacidosis	8.3 (4)	8.3 (4)
Blood ketone body increased	4.2 (2)	4.2 (2)
Acetonemia	2.1 (1)	2.1 (1)
Ketosis	2.1 (1)	2.1 (1)
Related to urinary tract infection	8.3 (4)	8.3 (4)
Cystitis	6.3 (3)	6.3 (3)
Pyuria	2.1 (1)	2.1 (1)
Asymptomatic bacteriuria	2.1 (1)	2.1 (1)
Related to genital infection	8.3 (4)	8.3 (4)
Fournier’s gangrene	2.1 (1)	2.1 (1)
Trichomoniasis	2.1 (1)	2.1 (1)
Vulvovaginal candidiasis	4.2 (2)	4.2 (2)
Vulvovaginal pruritus	2.1 (1)	2.1 (1)
Related to renal function	6.3 (3)	6.3 (3)
Pollakiuria	2.1 (1)	2.1 (1)
Polyuria	4.2 (2)	4.2 (2)
Related to hepatic function	2.1 (1)	0
Hepatic steatosis	2.1 (1)	0
Related to gastrointestinal system	20.8 (10)	4.2 (2)
Abdominal pain upper	4.2 (2)	0
Constipation	4.2 (2)	4.2 (2)
Dental caries	4.2 (2)	0
Diarrhea	8.3 (4)	0
Hematochezia	2.1 (1)	0
Nausea	4.2 (2)	0
Hemorrhoidal hemorrhage	2.1 (1)	0

AEs, adverse events; ADRs, adverse drug reactions.

^a^ AEs, including those after dose escalation to luseogliflozin 5 mg, were assessed throughout the study period (periods I and II), excluding those that occurred during placebo administration in the PL group (period I).

AEs associated with special interest incidences between pediatric and adult populations are compared in Table 7. Several categories of special interest-related AEs were absent in children, and none demonstrated clinically meaningful differences in incidence between age groups.

**Table 7.** Comparison of AEs of special interest in pediatric and adult population (safety analysis set)

AEs related to special interest	Luseogliflozin^a^	
	pediatric (48) % (*n*)	adult (1,262) % (*n*)
AEs related to hypoglycemia	2.1 (1)	3.3 (42)
AEs related to pollakiuria	8.3 (4)	3.3 (42)
AEs related to circulating plasma volume	0	1.6 (20)
AEs related to decreased blood pressure	0	0.6 (8)
AEs related to ketoacidosis	8.3 (4)	3.0 (38)
AEs related to urinary tract infection	8.3 (4)	3.1 (39)
AEs related to genital infection	8.3 (4)	1.3 (16)
AEs related to benign or malignant tumor	0	2.2 (28)
AEs related to cardiovascular system	0	4.1 (52)
AEs related to bone metabolism	0	1.9 (24)
AEs related to renal function	6.3 (3)	12.8 (161)
AEs related to hepatic function	2.1 (1)	3.2 (40)
AEs related to gastrointestinal system	20.8 (10)	19.1 (241)

AEs, adverse events.

^a^ AEs, including those after dose escalation to luseogliflozin 5 mg, were assessed throughout the study period (periods I and II), excluding those that occurred during placebo administration in the PL group (period I).

## Discussion

This 52-week phase 3 study, composed of a 12-week randomized, double-masked, placebo-controlled phase (period I) and a subsequent 40-week open-label extension (period II), evaluated the efficacy and safety of once-daily luseogliflozin 2.5 mg, with optional dose escalation to 5 mg, in Japanese children and adolescents with T2D. During period I, luseogliflozin produced clinically meaningful improvements in glycemic control, and its glucose-lowering effect was sustained for 52 weeks, confirming the durability of treatment benefits. The efficacy of luseogliflozin was consistent across subgroups, irrespective of baseline characteristics such as age, sex, or concomitant metformin use.

Compared with prior pediatric clinical trials of SGLT2 inhibitors [17–20], this study demonstrated more pronounced reductions in HbA1c, whereas the placebo group showed only modest HbA1c increases. These differences may be attributed to the exclusion of insulin and other oral antidiabetic agents besides metformin, in contrast to phase 3 studies of empagliflozin [17], dapagliflozin [18], and canagliflozin [19, 20], which required combination therapy. The inclusion of treatment-naïve participants and a relatively homogenous Japanese population, along with higher baseline HbA1c levels, may have contributed to the observed magnitude of effect. Furthermore, centering on a homogeneous Japanese population may have underscored treatment characteristics that are less readily apparent in more heterogeneous populations. These findings suggest that SGLT2 inhibitors serve as a first-line pharmacologic option for pediatric T2D. In this regard, some differences exist in the treatment paradigm in Japan compared with several other regions. Notably, in Japan, metformin is not uniformly designated as a mandatory first-line agent, including in adult clinical practice. Moreover, even in patients with elevated HbA1c levels (e.g., ≥8.5%), insulin therapy initiation depends on the clinical context. Within this framework, SGLT2 inhibitors may justifiably be considered a flexible therapeutic option.

Beyond HbA1c, favorable changes were observed in FPG, HOMA-R, and GA. Although luseogliflozin has been associated with significant weight reduction in adults [21], body weight, BMI, BMI standard deviation score, and POW reductions in this pediatric cohort were modest and not statistically distinct. Based on studies for other agents indicating that differences between adult and pediatric patients are potentially attributable to physiological growth during adolescence [13], the weight-reducing effect of luseogliflozin may also have been less apparent in pediatric patients for the same reason. Consistent with these findings, the T2NOW trial of dapagliflozin in children and adolescents with T2D also reported no significant body weight reductions [22].

The safety findings in this study align with the established safety profile of SGLT2 inhibitors in adults. The most frequently reported ADRs included cystitis, constipation, vulvovaginal candidiasis, elevated blood ketone levels, and polyuria – all known class effects. No deaths occurred. Three SAEs were reported, comprising one case of worsening primary disease and two anticipated events. Aside from transient increases in ketone bodies, no clinically relevant abnormalities were detected in laboratory or vital sign measurements. Evaluation of bone metabolism and growth-related parameters – including intact parathyroid hormone, bone-specific alkaline phosphatase, type I collagen N-telopeptide, 1,25-dihydroxyvitamin D, and 25-hydroxyvitamin D – showed no significant posttreatment changes (online suppl. Table 4). Similar findings were reported in a dapagliflozin study, which demonstrated that height, weight, BMI, Tanner stage, growth markers, and bone biomarkers remained unaffected in pediatric patients with T2D [23].

Urinary and genital infections are recognized class effects of SGLT2 inhibitors, linked to increased urinary glucose excretion, and thus require close monitoring [24]. In this study, a rare case of Fournier’s gangrene occurred in a 13-year-old female patient, was confirmed clinically in collaboration with dermatology, and was successfully managed surgically. Diabetes and alcoholism are recognized as the most significant risk factors for Fournier’s gangrene, with additional factors including HIV infection, advanced age, smoking, obesity, atherosclerosis, and malnutrition [25]. In this case, despite evaluation of these known risk factors, no definitive contributing factors to the development of Fournier’s gangrene – other than diabetes – could be identified. Although the incidence of Fournier’s gangrene is extremely low, emerging evidence suggests a possible association with SGLT2 inhibitor use, prompting regulatory warnings in adults [24]. In the USA, the number of reported cases of Fournier’s gangrene increased markedly following the 2018 safety warnings regarding its association with SGLT2 inhibitors, with two cases reported in individuals under 18 years of age [26]. Therefore, comparable vigilance and risk management strategies are warranted in pediatric patients.

Overall, the safety profile of luseogliflozin in this study was consistent with known class effects of SGLT2 inhibitors in adults, and no pediatric-specific risks were identified. Observed adverse effects were manageable through existing risk mitigation strategies.

Several limitations should be acknowledged. As comprehensive genetic testing was not performed, atypical or rare diabetes could not be completely excluded. Additionally, this study could not separately evaluate the long-term efficacy of luseogliflozin at the 2.5-mg starting dose alone or the efficacy in patients who required dose escalation to 5 mg owing to the limited sample size; the small sample size also limited subgroup analyses and may have reduced power to detect uncommon safety signals. The influence of luseogliflozin on pubertal stage was not established, and the absence of direct growth assessments restricted conclusions regarding long-term effects on growth-related parameters. Additionally, HOMA-R was assessed only at week 12, without repeated longitudinal assessments, which limits interpretation regarding sustained effects on insulin resistance independent of glycemic improvement. The 52-week study duration was insufficient to assess long-term glycemic control, growth outcomes, or diabetes-related complications. The lack of a comparator group and the exclusion of glucagon-like peptide-1 receptor agonists and insulin reduce the generalizability of these findings. Finally, interpreting the applicability of the findings to non-Japanese populations may require cautious interpretation.

In conclusion, luseogliflozin, administered at the same dosage used in adults, provided significant and sustained improvements in glycemic control compared with placebo and demonstrated a tolerable safety profile in Japanese children and adolescents with T2D. Considering its insulin-independent glucose-lowering mechanism via urinary glucose excretion, luseogliflozin represents a promising therapeutic option for pediatric T2D, particularly in cases characterized by obesity-related insulin resistance – a pathophysiological feature prevalent among Japanese youth.

## Acknowledgments

We thank the study participants and investigators of 58 sites: Saitama Medical University Hospital, Yokohama City University Medical Center, Niigata University Medical & Dental Hospital, Kurashiki Central Hospital, Tokushima University Hospital, Ibaraki Seinan Medical Center Hospital, Minami Akatsuka Clinic, Isesaki Municipal Hospital, Ota Memorial Hospital, Saitama Hospital, Nihon University Hospital, Kanazawa Medical Center, University of Yamanashi Hospital, Shizuoka Children’s Hospital, Daido Clinic, Omihachiman Community Medical Center, Izumi City General Hospital, Osaka City General Hospital, Takahashi Clinic, Tottori University Hospital, Hospital of the University of Occupational and Environmental Health, Fukushima Medical University Hospital, St. Marianna University Hospital, Matsumoto Medical Center, Osaka Metropolitan University Hospital, Kurume University Hospital, University Hospital Kyoto Prefectural University of Medicine, Nara Prefecture General Medical Center, Ehime University Hospital, Asahikawa Medical University Hospital, Tokuyama Central Hospital, Tokyo Women’s Medical University Hospital, Jichi Medical University Saitama Medical Center, Steel Memorial Muroran Hospital, Gunma University Hospital, Hamamatsu University Hospital, Saitama Children’s Medical Center, Shiga University of Medical Science Hospital, Fukuoka University Hospital, Kawasaki Municipal Hospital, Hiroshima Prefectural Hospital, Taniyama Seikyo Clinic, Teine Keijinkai Hospital, Fukuoka Children’s Hospital, Social Medical Corporation Bokoi Tenshi Hospital, National Defence Medical College Hospital, Shiga Medical Center for Children, Nippon Medical School Musashikosugi Hospital, Shikoku Medical Center for Children and Adults, Japanese Red Cross Asahikawa Hospital, Japanese Red Cross Kitami Hospital, Kokura Medical Center, Kawaguchi Municipal Medical Center, Joo nico nico clinic, Sendai City Hospital, Tsuchiura Kyodo General Hospital, National Hospital Organization Nishi Beppu Hospital, and Hyogo Prefectural Kobe Children’s Hospital. We thank the former employees of Taisho Pharmaceutical Co., Ltd., for their support – Takako Ishizaki, Erina Yoshida, and Katsunori Tsukahara – for study design or editorial assistance. We also thank Maruzen-Yushodo Company, Limited (https://yushodo.maruzen.co.jp/), for the English language editing and publication support.

## Statement of Ethics

This clinical trial was conducted in accordance with the Declaration of Helsinki. This study protocol was reviewed and approved by Ethics Committees at each of the participating sites. This full list of participating sites and Ethics Committees have been provided in online supplementary Table 1. Written informed consent for study participation was obtained from the parents/guardians of all participants, and pediatric patients provided assent.

## Conflict of Interest Statement

Toru Kikuchi reports receiving consulting fees from Novo Nordisk Pharma Ltd., MTI Ltd., Sanofi K.K., and Taisho Pharmaceutical Co., Ltd. Hidefumi Nakamura has received research grants from the Japan Agency for Medical Research and Development and the Ministry of Health, Labour and Welfare. He has also received consulting fees from LinDo K.K., Sato Pharmaceutical Co., Ltd., Taisho Pharmaceutical Co., Ltd., Tohoku University, and Pfizer R&D Japan G.K. Hideo Umeuchi, Iwao Kitajima, and Hironori Yamasaki are employees of Taisho Pharmaceutical Co., Ltd. Tatsuhiko Urakami reports receiving consulting fees from Taisho Pharmaceutical Co., Ltd.

## Funding Sources

This study was conducted as a sponsor-initiated clinical trial by Taisho Pharmaceutical Co., Ltd., which was involved in various aspects of the research, including study design, data analysis and interpretation, report preparation, and the decision to submit the manuscript for publication.

## Author Contributions

Study design, conduct, and data collection: T.K., H.N., H.U., I.K., H.Y., and T.U. Writing and/or reviewing the manuscript: T.K., H.N., H.U., and T.U. Data analysis and interpretation, and final approval of the manuscript for submission: all authors.

## Data Availability

The data that support the findings of this study are not publicly available because they contain proprietary information owned by Taisho Pharmaceutical Co., Ltd., but are available from the corresponding author upon reasonable request and subject to permission from Taisho Pharmaceutical Co., Ltd.
